# Supplementary material for: Barriers to Hepatitis B Screening and Prevention for African Immigrant Populations in the United States: A Qualitative Study
Source: Viruses. 2020 Mar 11;12(3):305. doi: 10.3390/v12030305 (PMC7150884; doi:10.3390/v12030305)
Supplement: Supplementary file 1 [file viruses-12-00305-s001.pdf]

Supplementary Materials:

Code	Definition/Description
Perceptions and awareness	Awareness and discussion level about HBV compared to other illnesses, in the community
Advisor perception of health issues	Describes or lists the health issues faced by AI communities, from the perspective of the advisory committee member. In other words, it describes the health issues that the advisor feels are most prevalent or important.
Community perception of health issues	Describes or lists the health issues faced by AI communities, from the perspective of the community. In other words, it describes the health issues that advisors hear the communities talk about the most.
Community awareness of HBV	From the advisor's perspective, it describes the level of awareness about HBV in the communities they serve, on its own, or in comparison (of awareness level) with health issues.
Religious beliefs about illness	Discusses the role that religious beliefs play in the communities' perceptions about illness.
Religion and health care providers	Specifically, discusses the impact of illness-related beliefs on the attitude towards OR behavior of seeing a health care provider.
Religion and preventive screenings	Specifically, discusses the impact of illness-related beliefs on the attitude towards OR behavior of getting tested for diseases (preventive medicine).
Role of religious leaders	Role of religious leaders in health care seeking, illness-beliefs, or preventive screenings. Can be positive or negative.
Cultural differences not related to health	Any text that describes cultural differences among different communities not related to health OR general cultural differences mentioned.
Cultural beliefs about illness and medicine	Discusses the role that culture play in the communities' perceptions about illness. Any text that is general, and does not fit into one of the religious sub-codes would be coded here.
Culture and health care providers	Specifically, discusses the impact of culture on the attitude towards OR behavior of seeing a health care provider.
Culture and preventive screenings	Specifically, discusses the impact of culture on the attitude towards OR behavior of getting tested for diseases (preventive medicine).
Mistrust of medical profession	Describes mistrust or skepticism of western medicine, specifically as a barrier to preventive medicine seeking, or to seeking HBV screening or treatment/care.
Use of traditional medicine	Describes preference for using traditional medicine, in general, or for HBV-related services – especially as an alternative to using western medicine.
Barriers	Any text related to barriers to health care
Barriers to health care access	Describes or lists barriers to accessing health care that AI communities face. For first run-through of coding, **ALL** barriers will go into one general code **EXCEPT** fear-related barriers. We will break them into sub-codes after the first pass through coding.
Fear	Any mention of fear, as it serves as a barrier to HBV screening, preventive care, or care seeking in general. This would include fear of discrimination, deportation, confidentiality breaches, etc.
Health information resources	Any text related to information resources

Most used health information resources	Resources that community members use most often to get health information; any mention of languages that these health resources are in would go here.
Most trusted health information resources	Describes resources that community members trust to get health information.
HBV needs for community providers	Any text describing needs of those serving (working in) the communities
Information needs	Information that would help providers educate the community about HBV.
HBV resource needs	HBV resources that would be helpful for providers to have, so that they can meet the HBV needs in their communities.
HBV training needs	Types or topics for training that would be useful for providers to have, so that they can meet the HBV needs in their communities.
Patient education materials for community	HBV resources that could be given out to the community, that would be effective in improving knowledge and awareness at the community level.
Literacy issues or strategies	Any text describing literacy issues or strategies to overcome them
Literacy issues	Describes or lists literacy issues that could be a barrier to HBV education or development of HBV educational resources.
Generational differences in literacy	Describes differences in generational literacy levels for example- older populations vs. younger population literacy levels.
Strategies to overcome literacy issues	Description of strategies that could help overcome literacy issues
Cultural sensitivities	Possible cultural issues that should be noted when developing new educational resources.
HBV myths and stigma	Any text describing HBV-related myths, misinformation or feelings of stigma
HBV myths and misinformation	Description of myths or misinformation surrounding HBV causes, transmission, prevention, outcomes, treatments, severity, etc.
HBV-related stigma	Description of any feelings of stigma that people with HBV might describe, or that might serve as a barrier to HBV screening.
Needs and strategies (non-resource)	Any text describing non-resource needs of the community, as well as non-literacy strategies that can help in the community

Interview Guide

1. Please tell me about the African immigrant population(s) that you work in.
    a. Prompts may be used for the following, if needed: What countries or regions they are from in Africa, what their insurance status is, what their overall socio-economic status is (education, employment), how long they have been in the U.S. in general (i.e. newer vs. longer-term)
2. Do you also work directly with professionals who serve African immigrant communities – and if so, with whom do you work?
    a. Prompts may be used for the following, if needed: social service providers, patient navigators, clinicians, translators, religious leaders, community leaders, health educators
3. What are the overall health issues you think African immigrants in the communities you serve are facing?
4. What health issues do African immigrants know, or talk, the most about?
    a. Have you ever heard concerns expressed by your community members about hepatitis specifically?
5. Are you aware of how cultural beliefs may influence attitudes and behaviors about sickness (illness), among the African immigrant communities you serve?
    a. Can you provide some examples?
    b. Do you think cultural beliefs play a role in influencing decisions to go to health care providers? And if so, can you explain how?
    c. Do you think cultural beliefs play a role in influencing decisions to get tested for specific diseases? And if so, can you explain how?
6. Are you aware of how religious beliefs might influence attitudes and behaviors about sickness (illness), among the African immigrant communities you serve?

a. Can you provide some examples?

b. Do you think religious beliefs play a role in influencing decisions to go to health care providers? And if so, can you explain how?

c. Do you think religious beliefs play a role in influencing decisions to get tested for specific diseases? And if so, can you explain how?

7. What are some of the barriers to accessing health care in the African Immigrant communities you serve? *Note to Interviewer: Please ask them to explain a few of the barriers they consider to be most important in more detail, not just list them*

a. Prompts may be used for the following, if needed: health insurance/cost, difficulty navigating the health care system, residency status, mistrust of Western medicine, skepticism of Western medicine, not wanting anyone to know personal or family information

b. What do you know about their English proficiency? Do they need translation services?

8. I want to talk a bit about how people in the African immigrant community you serve learn about health issues:

a. From who do people get health information?

i. Prompts may be used for the following, if needed: interpersonal communication from health care workers, teachers, family members, friends, community organizations, professional organizations (like CDC), religious leaders, health care providers

b. What sources do people use to get health information?

i. Prompts may be used for the following, if needed: radio, TV, newspapers, brochures

c. Do any materials exist in the languages you need? Are they written?

        i. Prompts may be used for the following, if needed: Brochures and fact sheets, radio shows, newspaper ads

    d. Who or what do you think are the most trusted sources for getting health information among the African immigrant communities you serve?
    e. Are there any literacy issues we should be aware of?
    a. How do you generally overcome literacy issues when providing health education?
        i. Prompts may be used for the following, if needed: using images/graphics, using radio/audio/video resources

I would now like to ask you a few hepatitis B-specific questions:

9. What are your thoughts on the overall level of awareness and knowledge about hepatitis B in the African immigrant communities you serve?
    b. How does the level of awareness compare to that of other diseases – for example, HIV, diabetes, high blood pressure?
        i. prompt if needed: Do people know more or less about HBV than other diseases?
    c. Do you think, or have you heard, of any hepatitis B-related myths that are common?
        i. prompt if needed: myths about transmission, prevention or cure of HBV
    d. Do you think, among individuals that have an awareness of HBV, that there is specific hepatitis B-related stigma that serves as a barrier to getting tested for hepatitis B?
10. What kind of information would be most helpful to *you* in addressing hepatitis B among African Immigrant communities you serve?
    e. Prompts may be used for the following, if needed: basic hepatitis B information, information on how to dispel myths, resources for local testing sites, resources to refer infected individuals, information on how to conduct HBV education and testing

11. What kind of training would be most helpful to you in addressing hepatitis B among African Immigrant communities you serve?
    f. Prompts may be used for the following, if needed: webinars, in-person trainings, conferences, peer mentoring
12. What kinds of resources would be most helpful to you in addressing hepatitis B among African Immigrant communities you serve?
    g. materials for you: PowerPoint slides to provide education, cheat sheets, algorithms, other ideas
